# Antigenic and molecular evidence of *Brucella* sp.-associated epididymo-orchitis in frugivorous (*Artibeus lituratus*) and nectarivorous (*Glossophaga soricina*) bats in Brazil

**DOI:** 10.3389/fvets.2023.1235299

**Published:** 2023-08-28

**Authors:** Laice A. Silva, Monique F. S. Souza, Camila G. Torquetti, Daniele C. O. Freitas, Larissa G. A. Moreira, Thaynara P. Carvalho, Clarissa H. Santana, Julia R. Thompson, Daniela C. O. Rosa, Tatiana R. Jesus, Tatiane A. Paixão, Renato L. Santos

**Affiliations:** ^1^Departamento de Clínica e Cirurgia Veterinárias, Escola de Veterinária, Universidade Federal de Minas Gerais, Belo Horizonte, Minas Gerais, Brazil; ^2^Sete Soluções e Tecnologia Ambiental, Belo Horizonte, Minas Gerais, Brazil; ^3^Departamento de Patologia Geral, Instituto de Ciências Biológicas, Universidade Federal de Minas Gerais, Belo Horizonte, Minas Gerais, Brazil

**Keywords:** *Brucella*, brucellosis, epididymitis, orchitis, bats, *Artibeus lituratus*, *Glossophaga soricina*

## Abstract

This study included 47 free-ranging bats from the State of Minas Gerais, Brazil. Six bats (12.8%) had genital inflammatory lesions, and two of them (one *Artibeus lituratus* and one *Glossophaga soricina*, a frugivorous and a nectarivorous, respectively) were diagnosed with *Brucella* sp. infection through PCR, and antigens in intralesional macrophages were detected using immunohistochemistry.

## Introduction

*Brucella* are gram-negative facultative intracellular bacteria belonging to the class of Alphaproteobacteria. Some species of the genus are causative agents of brucellosis, a disease associated with economic losses in livestock and public health issues (1). Classical *Brucella* species, originally isolated from terrestrial mammals—mostly domestic animals, were taxonomically classified according to their pathogenicity and host specificity, including *B*. *abortus* (cattle), *B*. *melitensis* (small ruminants), *B*. *suis* (pigs), *B*. *ovis* (sheep), *B*. *canis* (dogs), and *B*. *neotomae* (wood desert rats) (1). More recently, *B*. *microti* was isolated from the common vole (2), *B*. *pinnipedialis* and *B*. *ceti* were isolated from pinnipeds and cetaceans, respectively (3), *B*. *papionis* was isolated from baboons (4), and *B*. *inopinata* was isolated from a human breast implant (5), and both were recognized as new species. Atypical *Brucella* spp. have also been isolated from cold-blooded animals, such as amphibians (6), reptiles (7), and fish (8). Therefore, the recent expansion of the *Brucella* genus supports the notion that possibly many other *Brucella* species may infect wildlife (1).

Although *Brucella* spp. may have a broad cell and tissue tropism depending on the pathogen and host species (9), they are often associated with genital lesions in domestic animals (9, 10). For instance, *B. abortus* is an important cause of abortion in pregnant heifers and cows (11), and it is also an important cause of orchitis and epididymitis in males (10). In contrast, *B. ovis* infection in sheep is primarily associated with epididymitis in rams (12), although it may also be a cause of abortion in pregnant ewes (10). Bats have been investigated as potential hosts for *Brucella* spp. (13–16). Importantly, anti-*Brucella* antibodies have been detected in vampire bats (*Desmodus rotundus*) in Brazil (17), and recently, a novel *Brucella* species has been identified infecting vampire bats in Costa Rica (18). Thus, the finding of genital lesions, which are known to be associated with *Brucella* infection in many animal species (10–12), in free-ranging bats prompted us to investigate the possibility of *Brucella* infection. Therefore, this study provides evidence of *Brucella* sp. infection associated with genital lesions in frugivorous and nectarivorous bats, *Artibeus lituratus* and *Glossophaga soricina*, respectively.

## Methods

**Figure 1:**
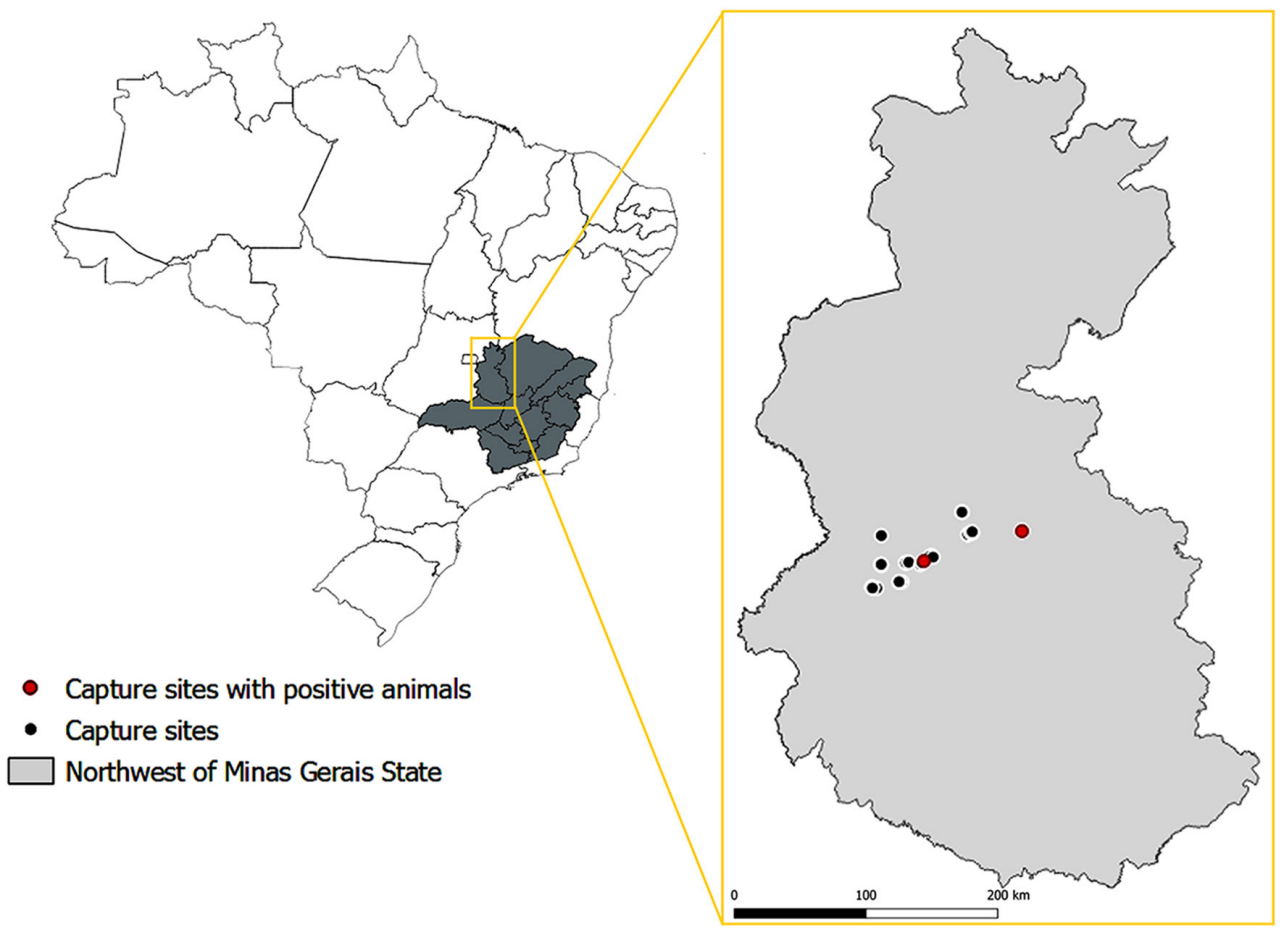
Location of sites where bats included in this study were captured, with the indication of locations where the capture of *Brucella* sp. infected bats took place. Source of maps: IBGE 2017. SRC: EPSG: 4674—SIRGAS 2000 (QGIS Version 3.28.2).

Samples of testes and epididymides were obtained from 47 free-ranging male bats belonging to eight species: *Carollia perspicillata* (*n* = 17), *Artibeus obscurus* (*n* = 11), *Glossophaga soricina* (*n* = 7), *Platyrrhinus lineatus* (*n* = 6), *Artibeus lituratus* (*n* = 3), *Dermanura cinerea* (*n* = 1), *Desmodus rotundus* (*n* = 1), and *Phyllostomus discolor* (*n* = 1). These bats were captured in the northwestern region of the State of Minas Gerais, Brazil (Figure 1) using mist nets. The bats were euthanized by anesthetic overdose. Capturing and sampling were performed as part of an environmental monitoring program that followed all applicable laws and regulations and was authorized by the *Instituto Estadual de Florestas* (IEF) under protocol number 024-002/2021. Tissue samples were fixed in 10% buffered formalin, embedded in paraffin, and processed for histological analysis.

DNA was extracted from paraffin-embedded samples of testes and epididymides of all bats using a commercially available kit (ReliaPrep FFPE gDNA Miniprep System, Promega, Madison, USA), following the manufacturer's instructions, and used as a template DNA for polymerase chain reaction (PCR) targeting *Brucella* spp. genomic sequences. PCR targeted the *Brucella* spp. *bcsp31* gene with primers 5′-TGGCTCGGTTGCCAATATCAA-3′ and 5′-CGCGCTTGCCTTTCAGGTCTG-3′, as previously described (19). PCR was performed with 2.5 μL of 10X Taq Buffer with KCl (Invitrogen, USA), 2.5 μL of 2 mM dNTP set solution (Invitrogen, USA), 1.5 μL of 25 mM MgCl_2_, 2.5 μL of each primer, 0.2 μL of Taq DNA polymerase (Invitrogen, USA), and 12 μL of template DNA samples with concentrations ranging from 64 to 200 ng/μL. The final reaction volume was 25 μL, with an expected product of 223 base pairs (bp). Amplification parameters were as follows: 94°C for 3 min; 40 cycles of 94°C for 30 s, 60°C for 30 s, and 72°C for 30 s, followed by a final extension at 72°C for 10 min. Amplified products were subjected to 1.5% agarose gel electrophoresis stained with SYBRSafe DNA Gel Stain (Invitrogen, USA) and examined in an ultraviolet light transilluminator.

Immunohistochemistry for *in situ* detection of *Brucella* sp. antigens was performed in samples from bats with genital lesions, as previously described (11), with modifications. Paraffin-embedded tissues were sectioned (4-μm-thick sections), dewaxed, hydrated, and washed three times in PBS. The sections were incubated with a 10% solution of hydrogen peroxide for 1 h for blocking endogenous peroxidase, rinsed in PBS, and then incubated with skimmed milk (25 μg/ml) for 45 min for blocking non-specific labeling. Sections were then incubated with a polyclonal anti-*Brucella* spp. antibody and diluted in the ratio of 1:1000 in 1% bovine serum albumin (BSA) for 1 h. The primary antibody employed in this study has been previously developed and characterized in our laboratory, as described in Xavier et al. (11), and it has been demonstrated to cross-react with various *Brucella* species (11, 20, 21). Sections were rinsed three times in PBS and incubated with a detection system (EnVision FLEX+; Dako, Carpinteria, CA, USA) for 30 min, followed by rinsing in PBS and development with chromogen 3′3-diaminobenzidine (DAB+ Substrate Chromogen; Dako) for 80 s, and sections were counterstained with Mayer's hematoxylin for 35 s. Positive controls included tissue sections from mice experimentally infected with *Brucella ovis*. Negative controls had the primary antibody replaced with sterile PBS.

## Results

**Figure 2:**
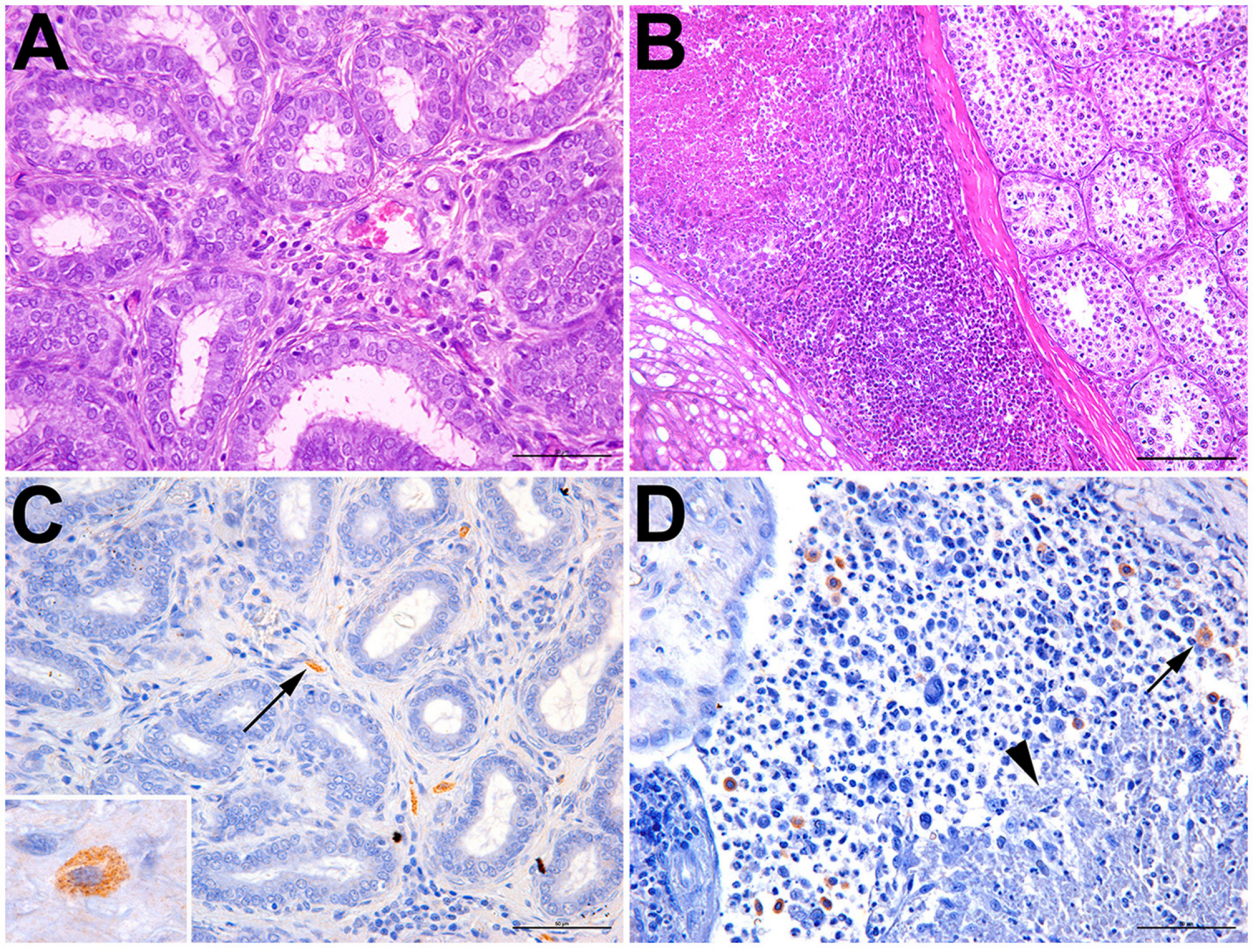
Epididymitis and orchitis in *Artibeus lituratus* (bat #17) and *Glossophaga soricina* (bat #10). **(A)** Bat #17; mild interstitial lymphohistiocytic epididymitis; hematoxylin and eosin, bar = 50 μm. **(B)** Bat #10; necrotizing and lymphohistiocytic epididymitis and periorchitis; hematoxylin and eosin, bar = 100 μm. **(C)** Bat #17; interstitial macrophages with cytoplasmic immunolabeled *Brucella* sp. (arrow); bar = 50 μm. Inset: immunolabeled cytoplasmic granular contents morphologically compatible with coccobacilli. **(D)** Bat #10; multiple macrophages with cytoplasmic immunolabeled *Brucella* sp. (arrow) adjacent to an area of necrosis (arrowhead); bar = 50 μm.

Six bats had inflammatory lesions in the testes and epididymis, including two *Artibeus obscurus* and one bat of each of the following species: *Carollia perspicillata, Glossophaga soricina, Artibeus lituratus*, and *Phyllostomus discolor* (Table 1). Histological changes were characterized by mild-to-severe multifocal to coalescing interstitial inflammatory infiltrate composed of lymphocytes and macrophages (Figure 2A). A necrotizing and histiocytic epididymitis was observed in one of the affected bats (bat #10; Figure 2B).

**Table 1:** Species, identification, geographic location, histopathology, immnunohistochemistry, and PCR of bats captured in Minas Gerais, Brazil, and analyzed in the study.

ID^*^	**Species**	**Latitude**	**Longitude**	**Histopathology**	IHC^*^	PCR^*^
1	*Carollia perspicillata*	−17.2094284013010	−46.7968984851780	Lymphohistiocytic epididymiti**s**	**–**	**–**
2	*Glossophaga soricina*	−17.2090227183659	−46.8099153647853	No histopathological changes	ND^*^	**–**
3	*Glossophaga soricina*	−17.2090227183659	−46.8099153647853	No histopathological changes	ND^*^	**–**
4	*Carollia perspicillata*	−17.2090227183659	−46.8099153647853	No histopathological changes	ND^*^	**–**
5	*Glossophaga soricina*	−17.2090227183659	−46.8099153647853	No histopathological changes	ND^*^	**–**
6	*Carollia perspicillata*	−17.1233716656568	−46.7795967688848	No histopathological changes	ND^*^	**–**
7	*Carollia perspicillata*	−17.1233716656568	−46.7795967688848	No histopathological changes	ND^*^	**–**
8	*Carollia perspicillata*	−17.1233716656568	−46.7795967688848	No histopathological changes	ND^*^	**–**
9	*Carollia perspicillata*	−17.1205612948793	−46.7662169475293	No histopathological changes	ND^*^	**–**
10	*Glossophaga soricina*	−17.1274627901764	−46.7921152531496	Necrotizing, neutrophilic and histiocytic epididymitis and periorchitis	**+**	**–**
11	*Artibeus obscurus*	−17.1303600564601	−46.8907098505170	No histopathological changes	ND^*^	**–**
12	*Carollia perspicillata*	−16.9799692981045	−46.2527613113983	No histopathological changes	ND^*^	**–**
13	*Artibeus lituratus*	−17.1303600564601	−46.8907098505170	No histopathological changes	ND^*^	**–**
14	*Carollia perspicillata*	−16.9799692981045	−46.2527613113983	No histopathological changes	ND^*^	**–**
15	*Artibeus obscurus*	−16.8938187491926	−46.5248956768913	No histopathological changes	ND^*^	**–**
16	*Carollia perspicillata*	−16.9799692981045	−46.2527613113983	No histopathological changes	ND^*^	**–**
17	*Artibeus lituratus*	−16.8938187491926	−46.5248956768913	Lymphohistiocytic epididymo-orchitis	**+**	**+**
18	*Platyrrhinus lineatus*	−16.9755501846642	−46.2527038133447	No histopathological changes	ND^*^	**–**
19	*Platyrrhinus lineatus*	−17.1150706248978	−46.6972015653591	No histopathological changes	ND^*^	**–**
20	*Glossophaga soricina*	−16.9755501846642	−46.2527038133447	No histopathological changes	ND^*^	**–**
21	*Carollia perspicillata*	−17.1150706248978	−46.6972015653591	No histopathological changes	ND^*^	**–**
22	*Artibeus obscurus*	−17.1308670927885	−46.7131237333135	No histopathological changes	ND^*^	**–**
23	*Platyrrhinus lineatus*	−17,0909766166065	−46.6616548068859	No histopathological changes	ND^*^	**–**
24	*Carollia perspicillata*	−17.1220231450009	−46.7053557324896	No histopathological changes	ND^*^	**–**
25	*Platyrrhinus lineatus*	−17.0909766166065	−46.6616548068859	No histopathological changes	ND^*^	**–**
26	*Phyllostomus discolor*	−17.1220231450009	−46.7053557324896	Lymphohistiocytic epididymitis	**–**	**–**
27	*Glossophaga soricina*	−17.0937648121599	−46.6709346406246	No histopathological changes	ND^*^	**–**
28	*Artibeus obscurus*	−16.8938187491926	−46.5248956768913	No histopathological changes	ND^*^	**–**
29	*Artibeus obscurus*	−17.2387727927019	−46.9109722226646	No histopathological changes	ND^*^	**–**
30	*Artibeus obscurus*	−17.0974575373092	−46.6559053774508	No histopathological changes	ND^*^	**–**
31	*Carollia perspicillata*	−17.2337085885199	−46.9322533158147	No histopathological changes	ND^*^	**–**
32	*Artibeus obscurus*	−17.2337085885199	−46.9322533158147	No histopathological changes	ND^*^	**–**
33	*Artibeus obscurus*	−17.2337085885199	−46.9322533158147	No histopathological changes	ND^*^	**–**
34	*Artibeus obscurus*	−17.2378555305486	−46.9303878104247	Lymphohistiocytic epididymitis	**–**	**–**
35	*Platyrrhinus lineatus*	−16.9948506990380	−46.4982208137282	No histopathological changes	ND^*^	**–**
36	*Carollia perspicillata*	−16.9948506990380	−46.4982208137282	No histopathological changes	ND^*^	**–**
37	*Carollia perspicillata*	−16.9948506990380	−46.4982208137282	No histopathological changes	ND^*^	**–**
38	*Carollia perspicillata*	−16.9887021448849	−46.4776516094202	No histopathological changes	ND^*^	**–**
39	*Artibeus lituratus*	−16.8938187491926	−46.5248956768913	No histopathological changes	ND^*^	**–**
40	*Glossophaga soricina*	−16.9863569703071	−46.4857380000083	No histopathological changes	ND^*^	**–**
41	*Carollia perspicillata*	−16.9825977824704	−46.4782517987072	No histopathological changes	ND^*^	**–**
42	*Desmodus rotundus*	−16.8938187491926	−46.5248956768913	No histopathological changes	ND^*^	**–**
43	*Artibeus obscurus*	−16.8938187491926	−46.5248956768913	No histopathological changes	ND^*^	**–**
44	*Platyrrhinus lineatus*	−17.1357163764718	−46.8899182767961	No histopathological changes	**–**	**–**
45	*Dermanura cinerea*	−17.1357163764718	−46.8899182767961	No histopathological changes	ND^*^	**–**
46	*Carollia perspicillata*	−17.1303600564601	−46.8907098505170	No histopathological changes	ND^*^	**–**
47	*Artibeus obscurus*	−17.1303600564601	−46.8907098505170	Lymphohistiocytic periorchitis	**–**	**–**

^*^ID#, identification number; IHC, immunohistochemistry; PCR, polymerase chain reaction; ND, not determined.

DNA samples extracted from paraffin-embedded testes and epididymides from all 47 bats were subjected to PCR targeting a *Brucella* spp. genomic sequence. Of the six bats, one bat (bat #17; *Artibeus lituratus*) had genital lesions tested positive by PCR, whereas none of the bats without genital lesions tested positive (Table 1).

*Brucella* spp. antigens were detected by immunohistochemistry in two bats, mostly associated with intralesional macrophages (Figures 2C, D; Table 1), including bat #17 (*Artibeus lituratus*), which also tested positive by PCR; and bat #10 (*Glossophaga soricina*). In some cells, the immunolabeled cytoplasmic contents of macrophages were morphologically compatible with coccobacilli (Figure 2C).

Considering the combined results of PCR and immunohistochemistry, in only two affected genera, *Glossophaga* and *Artibeus* (in total 21 bats), an estimated prevalence for that particular area [latitude extending from −16.8938187491926 to −17.2387727927019; and longitude from −46.2527038133447 to −46.9322533158147 (Figure 1)] was 9.52%, with a confidence interval between 1.18 and 30.38% (95% confidence level). Only the two genera that had positive animals were included in this estimate of prevalence since our data did not demonstrate the susceptibility of the other genera included in this study.

## Discussion

These are the first reported cases of *Brucella* sp. infection in *Artibeus lituratus* and *Glossophaga soricina*, which are frugivorous and nectarivorous bats, respectively. Importantly, molecular evidence of *Brucella* sp. infection, based on the same PCR protocol employed in this study along with real-time PCR, was reported in bats of the species *Myotis schreibersii* and *Myotis blythii* from Georgia, where four cases were identified among 236 bats included in that study (13). Importantly, in addition to PCR, our study provided a second line of evidence of *Brucella* sp. infection, which was the intralesional *in situ* detection of *Brucella* sp. antigens by immunohistochemistry. In these cases, immunolabeling of *Brucella* antigens was morphologically compatible with coccobacilli associated with intralesional macrophages in sites of epididymal and testicular inflammation (epididymitis and orchitis, respectively), which are common *Brucella*-induced lesions in other host species (10). This is a relevant finding since it strongly supports the hypothesis that *Brucella* sp. has pathogenic potential for these animals. Although previous surveys (14–16), including one previous survey in the State of Minas Gerais, Brazil (16), failed to demonstrate evidence of *Brucella* sp. infection in various species of bats from different parts of the world, a pioneering study has demonstrated serologic evidence of *Brucella* infection in vampire bats (*Desmodus rotundus*) in Brazil (17). Importantly, during the course of this study, infection of *Desmodus rotundus* vampire bats with a novel *Brucella* species was described, and the newly identified species was named *Brucella nosferati* (18). Unfortunately, only formalin-fixed samples were available in this study, which prevented us from attempting isolation and characterization of the *Brucella* sp. that infected these bats.

The criterion for investigating *Brucella* spp. infection in these cases was to find inflammation in the testis and epididymis. In many domestic animal species, *Brucella* has a well-established tropism for the genital system (9), and it is an important cause of epididymitis and orchitis in various species (10, 22). The recent expansion of the *Brucella* genus with the recognition of *Brucella* sp. infecting many wildlife species (1) and the finding of epididymitis and orchitis in these South American bats lead us to these diagnoses, which are relevant in the context of the expanding knowledge on brucellosis in wildlife and its zoonotic potential, with obvious relevance in the context of One Health. Importantly, the tropism of newly recognized *Brucella* spp. from wildlife is still poorly characterized (9). However, there are a few cases of genital infections with *B. ceti* affecting various cetacean species (23), including a well-characterized case of necrotizing epididymo-orchitis with intralesional labeling of *Brucella* sp. and isolation of *B. ceti* from the testis and epididymis of a harbor porpoise (*Phocoena phocoena*) (24).

Surveillance studies, such as the present study, are highly relevant since bats play an important ecological role by providing pest control, plant pollination, and seed dispersal (25). Many bat species have territorial behavior, so they tend to return to the same refuge. However, due to increasing anthropogenic activities and habitat losses, there is a tendency for agglomeration of bat populations in restricted areas (26). Furthermore, bats differ from other mammals of the same size in several ways, including their ability to disperse rapidly and widely, the highly gregarious nature of their social structures, and their long lifespans, which are features that may favor disease transmission and dissemination (27). Bats have been identified as reservoirs or disseminators of many zoonoses or diseases that may be transmitted to other animal species (27–29). Therefore, future studies should assess the zoonotic potential or risk to farm animals posed by bat-derived *Brucella* strains. Importantly, although brucellosis usually has high morbidity and low mortality and is often associated with reproductive failure (10), the emergence of certain infectious diseases may result in marked population decline as observed after the introduction of *Pseudogymnoascus destructans* in North America, which causes white-nose syndrome in bats, and resulted in the collapse of bat populations as one of the most devastating outbreaks affecting wildlife ever recorded (30).

## Conclusion

In conclusion, this study demonstrated *Brucella* sp. infection through two complementary diagnostic methods (immunohistochemistry and PCR) in *Artibeus lituratus* and *Glossophaga soricina*. Detection of *Brucella* sp. in these cases was associated with genital lesions. Importantly, demonstration of intralesional *Brucella* sp. in organs for which *Brucella* spp. have tropism in other host species (9) associated with lesions, namely epididymitis and orchitis, with a pattern of inflammation that have been classically recognized as *Brucella* spp.-induced lesions (10), supports the hypothesis that *Brucella* sp. is pathogenic for these species of bats. Unfortunately, the biological samples available in this study prevented us from isolating the organism, which could allow a proper identification at the species level.

## Data availability statement

The original contributions presented in the study are included in the article/supplementary material, further inquiries can be directed to the corresponding author.

## Ethics statement

The animal study was reviewed and approved by Instituto Estadual de Florestas (IEF) under protocol number: 024-002/2021.

## Author contributions

TP and RS: conceptualization, data curation, supervision, validation, and project administration. LS, MS, CT, DF, LM, TC, CS, JT, DR, and TJ: methodology. LS, MS, TP, and RS: formal analysis. LS, MS, DF, LM, TP, and RS: investigation. LS, MS, and RS: writing—original draft preparation and writing—reviewing and editing. All authors have read and agreed to the published version of the manuscript.
